# Characteristics of novel magnetic nanocomposites based on samarium orthoferrite

**DOI:** 10.1038/s41598-025-34318-3

**Published:** 2026-01-19

**Authors:** Kh. Roumaih, Sh. I. Hussein, S. A. Gad, A. M. Moustafa, I. A. Abdel-Latif

**Affiliations:** 1 Reactor Physics Department, Nuclear Research Center, Egyptian Atomic Energy Authority, Cairo, 13759 Egypt; 2 Solid State Physics Department, Physics Research Institute, National Research Centre, 33 El-Bohouth St, Dokki, 12622 Giza Egypt

**Keywords:** Multiferroic composites, orthorhombic structural, Cubic spinal structural, Ferromagnetic properties, Condensed-matter physics, Materials science, Physics

## Abstract

In this study, multiferroic composites MF/SmF (MF = NiFe_2_O_4_, CoFe_2_O_4_, ZnFe_2_O_4_, CdFe_2_O_4_, and SmF representing SmFeO_3_) were synthesized using the sol-gel method. X-ray diffraction (XRD), high-resolution transmission electron microscopy (HR-TEM), field emission scanning electron microscopy (FE-SEM), and Fourier transform infrared spectroscopy (FT-IR) were employed to examine the impact of SmF on the MF’s phase composition, microstructure, and degree of crystallinity. These analyses confirmed the formation of pure composites with two phases (orthorhombic + cubic), identified the chemical bonds present in the composites, and verified the nanoscale dimensions of all composites (17–92 nm). The composition element was determined through energy-dispersive X-ray analysis (EDXA). UV-vis absorption spectroscopy was used to assess the effect of SmF on the band gap energy of the MF, where the band gap energy of the SmF sample is 2.28 eV, CoF/SmF is 1.18 eV, and the NiF/SmF composite has an energy band gap of 1.8 eV. Electron paramagnetic resonance (EPR) spectroscopy, characterized by unpaired electron spins, and a vibrating sample magnetometer (VSM) were used to analyze the nanocomposites’ ferromagnetic characteristics. The exchange-coupled interaction between Sm and MF was confirmed by the lattice parameters and the functional groups present, as well as the energy gap for each compound and the magnetic parameters of SmF/FM nanocomposites.

## Introduction

Ferrite is a type of magnetic oxide based on iron. These materials exhibit opposing magnetic moments, enabling them to retain spontaneous magnetization. The properties of ferrite nanoparticles (NPs) are influenced by their chemical composition, particle size, and interactions with the surrounding matrix. As a result of these properties, ferrite NPs have a wide range of applications across various domains, particularly the biomedical and industrial sectors. Ferrites have different crystal structures based on the arrangement of oxygen and the surrounding metal cations. The main types of ferrites are spinel, perovskite (ortho-ferrites), garnets, and magneto-plumbites (hexagonal). Ultimately, the characteristics and applications of ferrites depend on their specific type.

 The magnetic properties of spinel ferrites are influenced by their composition, dopants, and synthesis conditions. Therefore, to obtain good magnetic properties such as saturation magnetic moment, coercivity, magnetic anisotropy, magnetostriction, etc., one must choose a new composition of ferrites based on the components’ intrinsic magnetic properties. Therefore, many researchers have worked on different ferrites through several experiments to obtain distinctive magnetic properties. Nickel, cobalt, zinc, and cadmium ferrites have unique magnetization values, such as saturation magnetism. Nickel and cobalt ferrites, which are inverse spinel ferrites, are found in high spin states. Their electronic configurations are t6 2ge2 g and t5 2ge2 g, respectively. Therefore, they are employed as ferromagnet insulators due to their high Curie temperatures (TC = 850 and 790 K, respectively) ^1,2^. Ferrite with a high cobalt content exhibits significant magnetocrystalline anisotropy, which leads to low permeability. Therefore, it is unsuitable for applications that require high permeability.

The crystal structure of zinc ferrite (ZFO) is cubic normal spinel. In addition to its advantageous characteristics of earth abundance, nontoxicity, ease of processing, and superior photochemical stability, the ZFO stands out among spinel ferrites because of its many uses, especially in photoelectric conversion and the production of hydrogen from water ^3^. Furthermore, ZFO is a promising semiconductor photocatalyst for photochemical processes because of its 1.9 eV ^4^ band gap, which allows it to absorb a sizable portion of the solar spectrum.

 Cadmium ferrite (CdF), a type of spinel ferrite compound, has been extensively studied because of its remarkable electromagnetic properties. It has high stability and good mechanical and magnetic properties. These characteristics position it as a strong candidate for use in soft magnets and low-loss materials at high frequencies^ 5^. It has been established that CdF exhibits cubic symmetry with a normal spinel structure ^6^.

On the other hand, SmFeO_3_ (SmF) is one of the unique orthoferrite family members. Due to its intriguing characteristics, this material has been extensively studied. It exhibits ferroelectricity, low dielectric loss, and the highest spin reorientation transition temperature, which is approximately 470 K. Additionally, it demonstrates magnetization reversal at low temperatures around 5 K. The material also exhibits significant piezoelectric properties at room temperature, and its dielectric constant varies with particle size^7–9^. Three definite magnetic interactions influence the magnetic behavior of SmFeO_3_: below 5 K, rare earth-rare earth interactions (R^3+^-R^3+^); at below 140 K, rare earth-iron interactions (R^3+^-Fe^3+^); and above 140 K, iron-iron interactions (Fe^3+^-Fe^3+^)^9^ ^,10^.

The combination of SmF with MF (NiF, CoF, ZnF, and CdF) presents an intriguing research domain as a multiferroic material. Because it combines the distinct qualities of spinel ferrite and samarium ferrite perovskite, a composite of these materials has advantages over each material alone. This results in synergistic effects that enhance performance in areas such as electromagnetic wave absorption and photocatalysis. While spinel ferrites offer superior magnetic properties and strong electrical resistivity, samarium ferrite perovskites offer variable electronic band gaps and surface characteristics. Greater charge separation gives the composite its increased photocatalytic activity, while the synergistic combination of magnetic and dielectric loss mechanisms from both components results in greater electromagnetic absorption ^11,12^. Composites of SmF and MF (spinel ferrite) are utilized for their rendering them advantageous for nanotechnology applications and magnetic recording, with the possibility for improved magnetic field regulation and control. Their combined structural and magnetic properties also make them candidates for other advanced applications, though specific details depend on the chosen M-element and composite synthesis method. Also, four distinct types of magnetic nanocomposites MF/SmF were synthesized. Nanocomposites MF/SmF have not been the subject of prior research reports. The objective is to examine the key interactions between MF and SmF and investigate their distinctive magnetic characteristics. This work will involve synthesizing the multiferroic material MF/SmF and examining its microstructure, optical characteristics, and magnetic properties. Additionally, we hope to help develop various innovative multifunctional device applications. In other words, this work aims to investigate the intricate magnetic behavior of multiferroic materials, specifically SmF/MF combinations. Of particular interest is how compounds such as perovskite affect the magnetic characteristics of different types of spinel.

## Experimental detail

### Synthesis of MF/SmF

Two steps must be taken to prepare the multiferroic nanocomposites MF/SmF. The first step was the preparation of SmF. MF nanoferrites grow over the SmF in the second step. The sol-gel technique was employed to synthesize nanocrystalline SmF. High-purity iron and samarium nitrates were dissolved in 200 milliliters of distilled water using magnetic stirring. Citric acid and one gram of polyethylene glycol were added to the solution. Ammonia was added while the mixture was continuously stirred for three hours at 85 °C to adjust the pH to 12. After this, the solutions were allowed to dry in an oven at 95 °C for 24 h. Once dried, the powders were annealed at 600 °C for three hours.

We will take NiF/SmF as an example to prepare the MF/SmF nanocomposite. Specific weights were used for nickel nitrate (Ni(NO_3_)_2_•6H_2_O) and ferric nitrate (Fe(NO_3_)_3_•9H_2_O), which were dissolved separately in distilled water and then combined. After that, the solution was mixed with a citric acid solution (C_6_H_8_O_7_•H_2_O) and left to stir for 120 min at room temperature while maintaining a steady speed. Add the ammonia solution dropwise until the pH hits 7 and 1.5 g of SmF nanoparticles, i.e., the FM is nearly twice as much as the SmF. The mixture was heated to create a gel, which then decomposed through spontaneous ignition, causing it to foam and puff up, resulting in a large volume of powder. The final powder was left to dry completely. To create the other nanocomposites (CoF/SmF, CdF/SmF, and ZnF/SmF), repeat the previous steps, adjusting the material weights. Throughout this process, we consistently maintained the ratio of MF to SmF at 2.5:1. Finally, the resulting powders (MF/SmF) were manually ground with an agate mortar, ensuring a fine and uniform consistency.

### Characterization

X-Ray Diffraction characterization of the prepared materials was done using a Bruker D 8 Advance Germany, equipped with Cu Kα radiation (λ = 1.54056Å) X-ray diffractometer in the 2θ range of 20–100° in the step scanning mode. The surface morphology and elemental composition were measured using field emission scanning electron microscopy (FESEM) images and energy dispersive X-ray analysis (EDX) spectra (ZEISS). This precise approach ensures a comprehensive understanding of the samples’ characteristics. A high-resolution transmission electron microscope (HR-TEM Model JEM-2100) is used for measuring the morphology and size of particles. Additionally, Fourier transform infrared (FTIR) spectra were recorded in the range of 400 to 4000 cm − 1 using equipment from Bruker Corp., located in Ettlingen, Germany.

The magnetic properties of the samples were examined at room temperature using a LakeShore 7410 vibrating sample magnetometer (VSM), which has a maximum magnetic field of 20 kOe. The electron paramagnetic resonance (EPR) spectra were recorded with a Bruker EMX electron spin resonance (ESR) spectrometer. Additionally, the optical properties of all samples were evaluated using a JASCO V-570 UV/Vis spectrophotometer from Japan equipped with an integrating sphere for diffuse reflection measurements ranging from 200 to 1500 nm.

## Results analysis and discussions

### Structural analyses

**Fig. 1:**
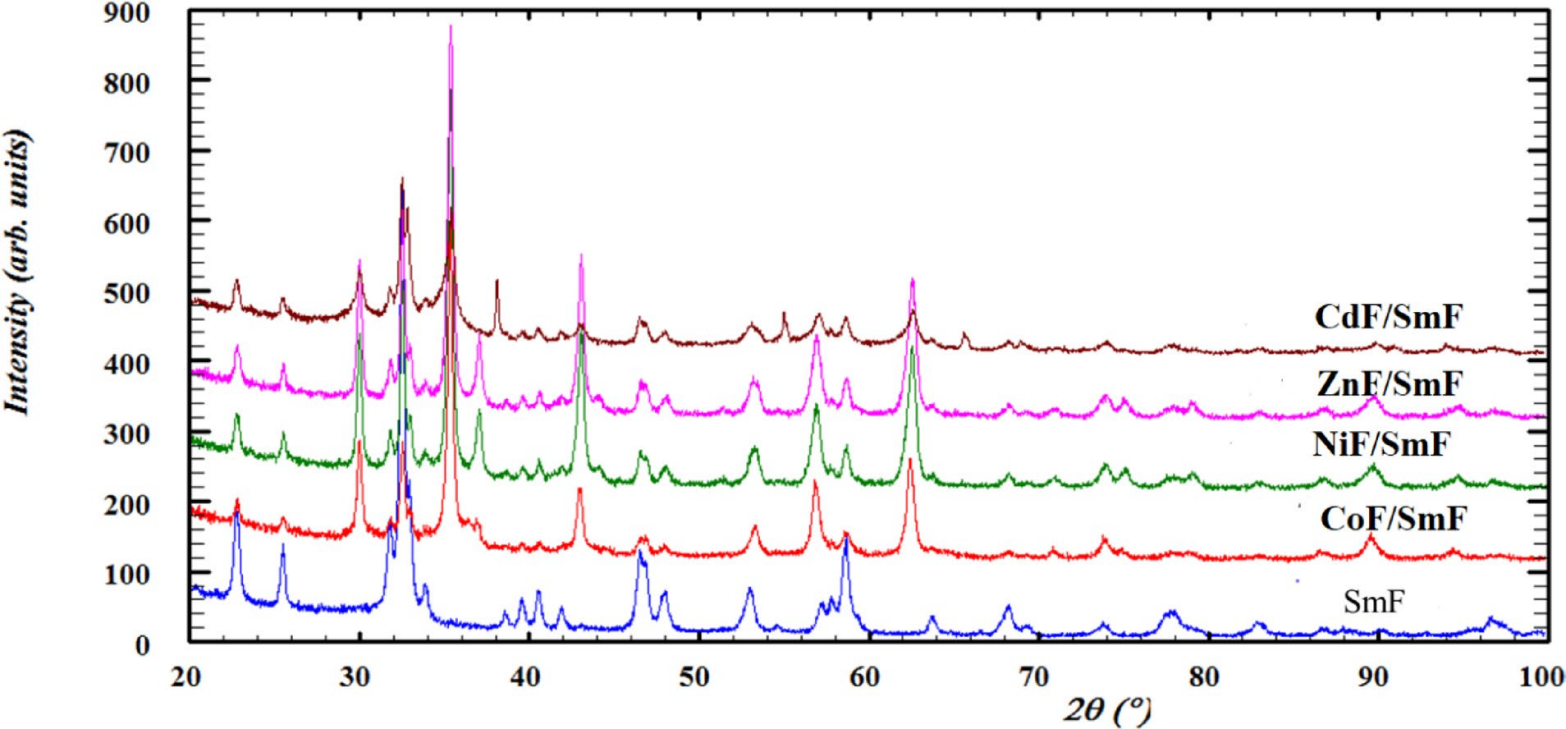
XRD for all samples SmFeO_3_ and MF/SmFeO_3_ nanocomposites.

Figure 1 shows the room-temperature XRD of the SmF and their composites. The figure shows no interdiffusion between the SmF and MF phases, which coexist in the composite as two distinct and separate phases. The XRD patterns of the composites displayed systematic variations in the intensities of the two phases (SmF, MF) upon incorporation. Using High Score Plus software (Fullprof program) ^13^ and ICDD data, we found no peaks associated with raw materials and other phases, indicating the formation of the perovskite structure SmF and the spinel structure of MF. The orthorhombic mono-phase perovskite structure SmF corresponds to ICDD card no. 98-002-7276. In contrast, the MF spinel structure varies based on the specific spinel compound. The MF compounds have the following reflections: (111), (022), (113), (222), (004), (133), (224), (115), (044), (244), (026), (335), (026), (444), (246), and (137). These reflections corresponded to the single phase of cubic spinel ferrite that matched with the ICDD card no. 98-008-4100 for NiF, 98-009-4871 for CoF, and 98-007-6981 for ZnF. Whereas the spinel CdF matched with ICDD card no. 96–591-0006, and there are extra peaks related to cadmium oxide with ICDD card no. 98-006-1554.

**Fig. 2:**
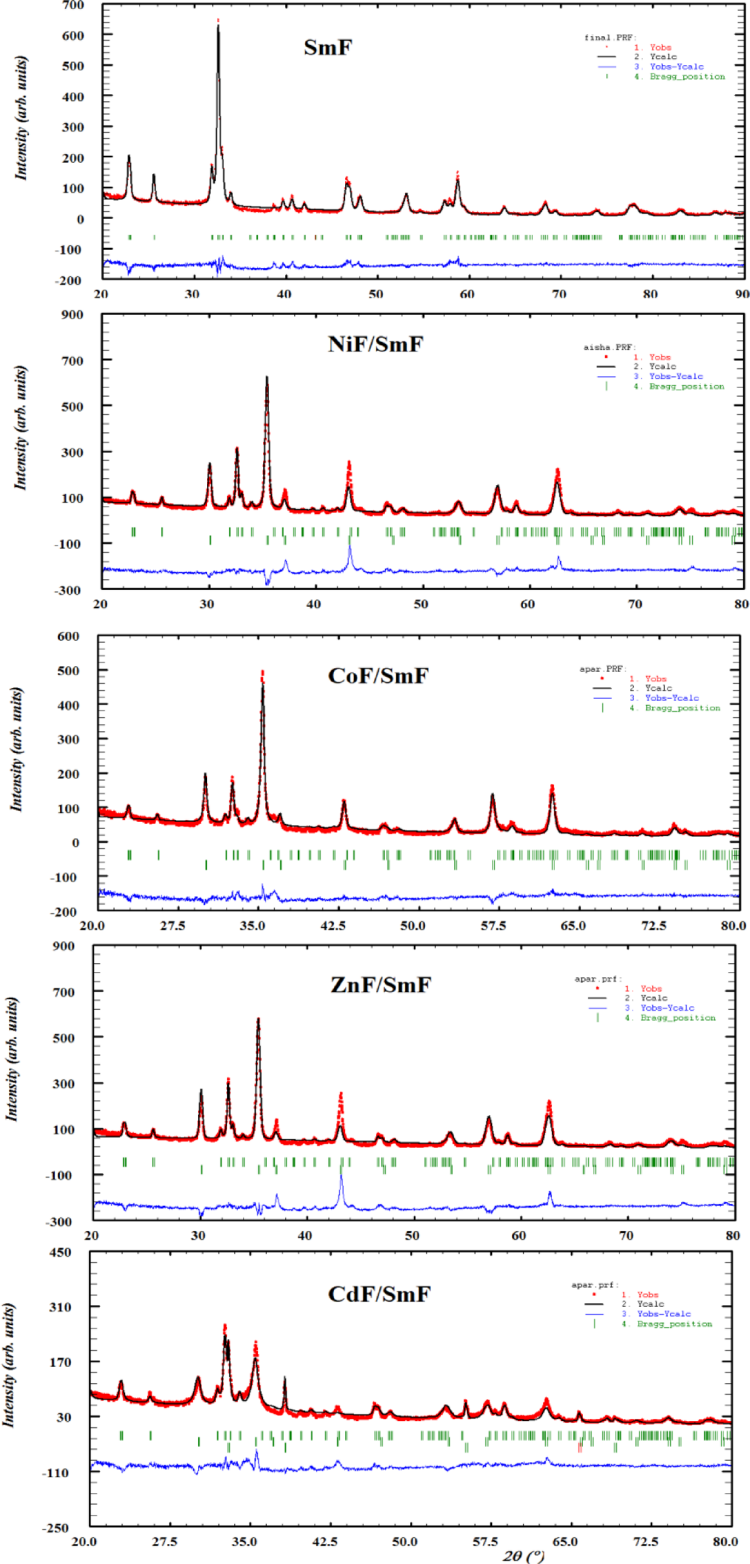
Rietveld refined XRD for SmFeO_3_ and MF/SmFeO_3_ nanocomposites.

The Rietveld refinement was performed on all samples to findthe crystal structure parameters of the prepared compounds (Fig. 2). For the perovskite structure (SmF), the atomic coordinates were taken using the space group pnma, in which O1 lies at the position 8d, O2 at 4c, Fe at 4a, and Sm at 4c. While for the spinel structure (MF), the space group was Fd-3 m, the O atom lies at the position 32e, while the tetrahedral and octahedral sites occupy the positions 8f and 16c, respectively. The peak shape was represented using a modified Thompson Cox-Hasting pseudo-Voigt ^14^ distribution function, which combines Gaussian and Lorentzian functions. The background data were described using fourth-order polynomials. The refinement process involved alterations to the lattice parameters, peak profile parameters (v, u, w, x, z), and atomic coordinates (x, y, z, B, and occupancy). The parameters (S) = Rwp/Rexp serve to assess the quality of the refinement. The refining process has been conducted to the point where the experimentally obtained pattern closely aligns with the theoretically derived XRD pattern. A goodness of refinement value indicates the caliber of the adjusted parameters. All the values of the refined parameters are tabulated in Table 1, which shows the unit cell volume of SmF with MF is lower than in the single phase without MF. This reduction may be attributed to the pressure exerted by the spinel phase within the composite. The unit cell volume of the spinel NiF, CoF, ZnF, and CdF aligns with previously published results ^15–18^. The refined values of the octahedral bond length of the spinel structures show that an increase in the octahedral bond length corresponds to a change in the tetrahedral metal cation. This increase in bond length could be caused by many factors:

**Table 1:** XRD parameters, size of the crystallites and values of the lattice strain of the cell of SmF/MF nanocomposites.

Parameter	SmF orth. phase				
	SmF	With NiF	With CoF	With ZnF	With CdF
**a (Å)**	5.6095(2)	5.6051(3)	5.4204(8)	5.6061 (5)	5.6064(3)
**b (Å)**	7.73333 (2)	7.7321(4)	7.7285(9)	7.7300(5)	7.7333(4)
**c (Å)**	5.4172 (1)	5.4164(3)	5.5964(8)	5.41163(4)	5.4104(4)
**volume (Å** ^**3**^ **)**	235.00 (1)	234.74 (2)	234.4 (6)	234.52(3)	234.57(2)
**Crystallite size (nm)**	48	47	52	46	31
**Microstrain(**abs.**)**	0.939(2)	1.191(3)	1.196(8)	1.417(3)	0.4(2)
	**MF Spinel phase**				
		**NiF**	**CoF**	**ZnF**	**CdF**
**a (Å)**		8.39681(9)	8.4052(2)	8.3938(2)	8.3898(3)
**volume (Å** ^**3**^ **)**		592.03(1)	593.81 (1)	591.74(2)	590.55(3)
**O-(M) octa (Å)**		2.033(3)	2.044(3)	2.049(6)	2.55(8)
**O-M tetra (Å)**		1.936(3)	1.980(3)	1.906(6)	1.561(8)
**Crystallite size (nm)**		66	33	92	17
**Microstrain (**abs.**)**		1.39	0.72(2)	0.72(2)	2.03(4)
**Rp**	38.7	44.9	57.0	47.4	41.7
**R**_**wp**_	40.4	39.2	49.0	48.0	40.8
**R**_**exp**_	31.3	32.9	37.7	28.1	41.1
**S** ^**2**^	1.662	1.422	1.69	2.925	0.9861
	**CdO phase a (Å)**				
					4.7077(1)
**Volume (Å** ^**3**^ **)**					104.338(3)
**Microstrain (**abs.**)**					0.321(9)
**Crystallite size (nm)**					140

- Variations in ionic radii brought on by oxidation states or the coordination environment,
- Modifications brought on by high-spin versus low-spin configurations.
- Structural deformities are brought on by variations in temperature or the substitutional impact.

The microstrain values of MF/SmF tend to increase to those of pure SmF. This could be because of a phase mismatch between the ferroelectric and magnetic phases in multiferroics, which can result in internal stresses. The lattice distortions caused by these stresses lead to an increase in microstrain. Because of the variations in lattice characteristics or thermal expansion, the interaction between the two phases may cause stress at the interfaces. However, the microstrain of SmF in the CdF composite is smaller than that of SmF alone, which could be due to the distortion of the CdO phase. In other words, the interaction between MF and SmF causes the ions to redistribute among the A and B sites of the interstitial space, changing the lattice parameter and characteristics of MF. Generally, the reciprocal relationship in the composites reveals flaws or disorders that impact the crystal’s chemical and physical characteristics. This mutual force has appeared in nanocomposites such as MFe/CuAl ^19^, NiF@MgF and ZnF@MgF ^20^.

Due to the broadening of the diffraction peaks shown in Fig. 1, the refined values of the crystallite size of SmF were in the nanoscale (Table 1). The crystallite size of the SmF, determined using a modified Thompson-Cox-Hastings pseudo-Voigt method ^14^ in the composites, decreased as the ionic radius of the tetrahedral sites in the spinel increased. The main reasons for this phenomenon are bigger cations, which increase the surface-to-volume ratio in crystallites due to their size and the resulting changes in crystal packing. As the crystallite size decreases, surface energy becomes much more significant. The system may choose smaller crystallites with larger grain boundaries to rectify the disparity between interior and surface atoms that are smaller and lower the total surface energy. There is no pattern for the spinel phase’s microstrain and crystallite size; therefore, the crystallite size is in the nanoscale.

Cation distribution is one of the most crucial factors that can significantly alter the ferrite material’s magnetic characteristics. The cation distribution for the MF compound is summarized in Table 2. Using this cation distribution, the degree of inversion (δ) can be calculated and expressed as (M_1−δ_ Fe_δ_)[M_δ_Fe_2−δ_]O_4_. Generally, when δ = 0, the spinel structure is considered normal, while at δ = 1, it is classified as inverted. For values of δ in between, the atoms A and B occupy both positions in varying quantities, and the spinel is called partially inverse. NiF and CoF exhibit partial inverse spinel characteristics, while CdF and ZnF are classified as normal spinel. These results align with the other literature [16, 17, 21, and 22].

**Table 2:** Cation distribution of MF.

Parameter	NiFe2O4	CoFe2O	ZnFeO4	CdFeO4
**Fe at octa ** **0.5** **0.5** **0.5**	1.97387	1.90829	2	2
**M at octa ** **0.5**,** 0.5 0.5**	0.02613	0.09171	0	0
**Fe at tetra ** **0.125**,** 0.125 0.125**	0.02613	0.09171	0	0
**M at tetra ** **0.125**,** 0.125 0.125**	0.97387	0.90829	1	1
**Degree of inversion δ**	0.02613	0.09171	0	0

### Surface morphology and EDX analysis

**Fig. 3:**
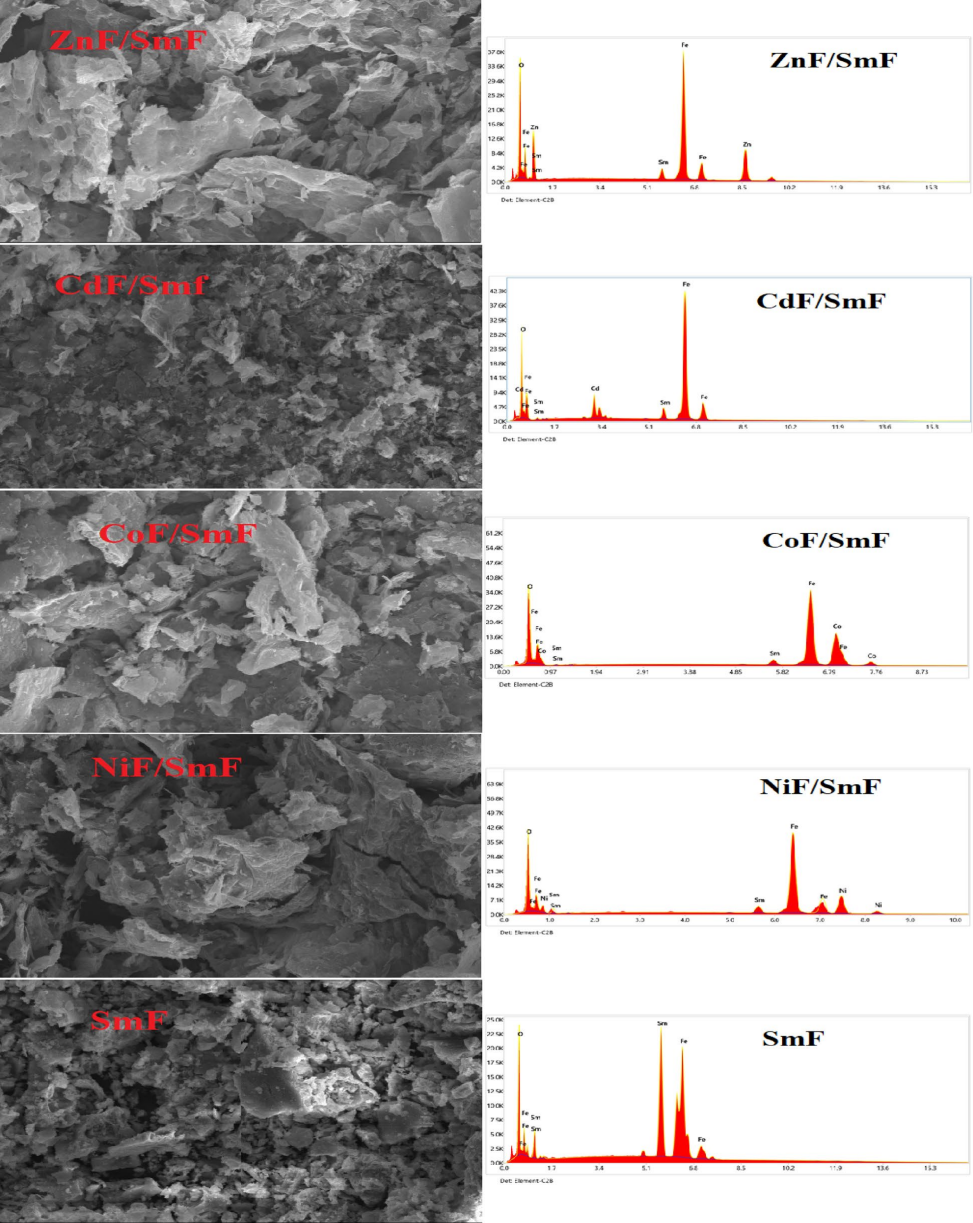
FESEM images and EDX spectra for SmFeO_3_ and MF/SmFeO_3_ nanocomposites.

The surface morphology of each sample was determined by analyzing the FE-SEM images. Figure 3 shows the FE-SEM images of all nanocomposites MF/SmF. One can observe the clearly defined grains and grain boundaries in the samples. Grain size and pore number vary with different MFs. The grain morphology of the MF/SmF nanocomposites is similar to that of other SmF compounds ^23–25^. The elemental composition was ascertained by energy-dispersive X-ray analysis (EDX) measurements, as illustrated in Fig. 3. The spectra confirm the presence of all elements in the sample. Specifically, peaks corresponding to Sm, Fe, Cd, Co, Ni, Zn, and O are observed in the EDX spectra of the MF/SmF nanocomposites. This indicates a homogeneous mixing of all atoms within the MF/SmF nanocomposites.

**Fig. 4:**
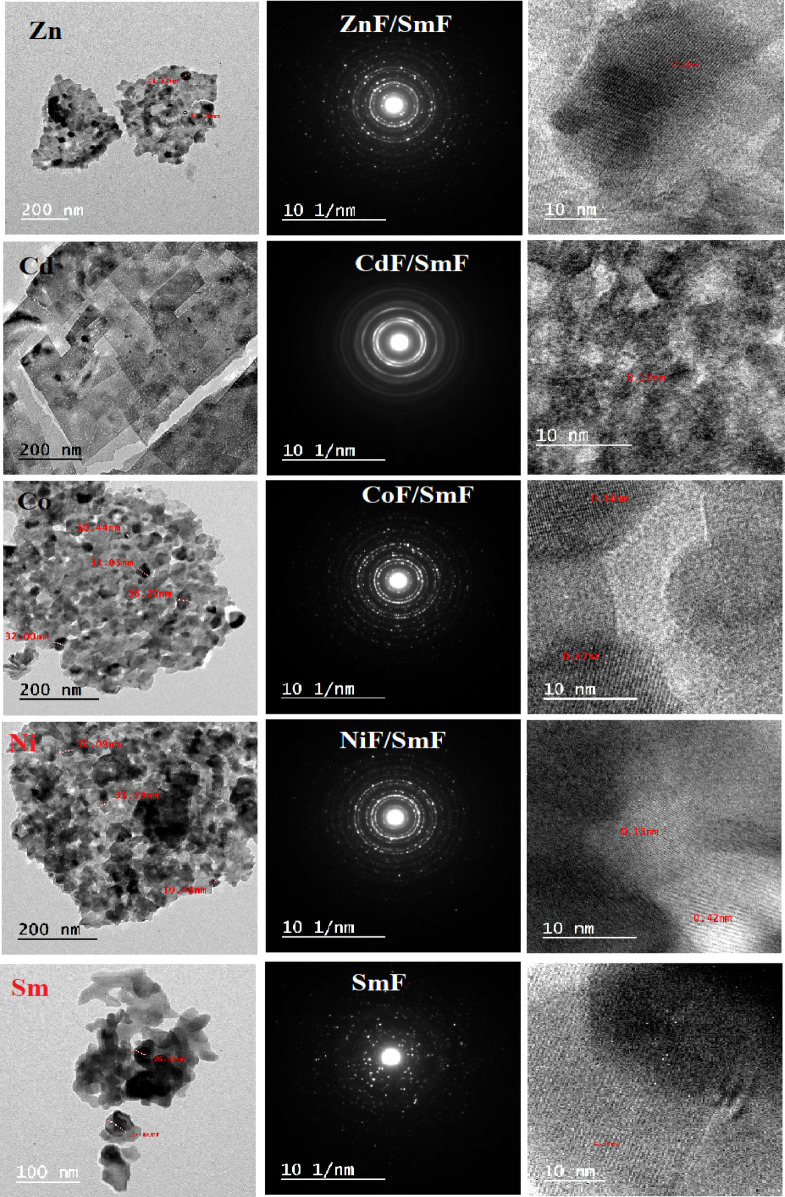
TEM micrograph images, HR-TEM the diffraction mode and SAER pattern for SmFeO_3_ and MF/SmFeO_3_ nanocomposites.

Figure 4 illustrates the morphology and crystallinity of MF/SmF nanocomposites. The size and crystallinity of the nanoparticles and the electron diffraction patterns have been confirmed. The TEM images show the shapes of the particles, except for CdF/SmF, which exhibits a nanoflake structure. According to Fig. 4, the sizes of the particles are as follows: SmF ranges from 26 to 37.6 nm, NiF/SmF from 19 to 31.9 nm, CoF/SmF from 10 to 36 nm, ZnF/SmF from 10.6 to 41.8 nm, and CdF/SmF from 5.4 to 10.7 nm. The particle size, however, varies due to several factors, such as the preparation method, heat treatment, and the associated compound or element. The difference in particle (TEM) and crystallite sizes (XRD) implies several particle domains in MF/SmF. According to the standard deviation of particle size, which seems to deviate from the XRD results, there is a discrepancy in the particle measurements. In other words, the particle count may be biased towards larger particles for SmF and smaller particles for MF.

### FT-IR analysis

**Fig. 5:**
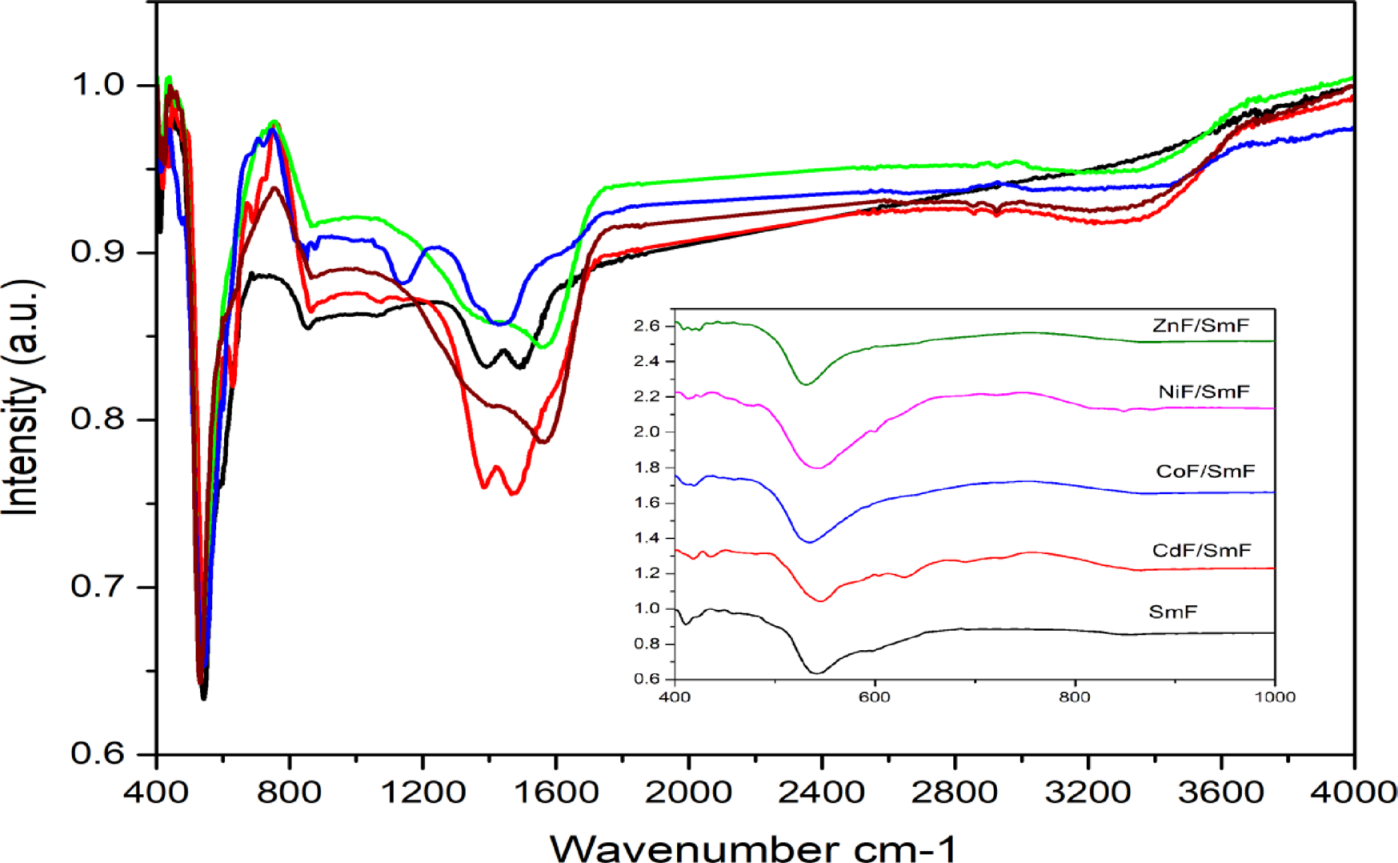
FTIR spectra for SmFeO_3_ and MF/SmFeO_3_ nanocomposites.

The IR spectra usefully revealed the chemical reaction, bonding vibrations (vibrational modes), ionic locations, the distribution of cations, and the deformation aided by substituent atoms in the ferrite nanocrystal. Two fundamental bands (ν1 and ν2) and four major absorption bands are typically visible in normal and inverse cubic spinel phases. For spinel ferrites, two bands have been reported by Waldron ^26^ in the range 1000–300 cm^− 1^. Figure 5 shows the FT-IR spectra of SmF and MF/SmF nanostructures. In the range of 1000 to 400 cm ^− 1^, there are sharp peaks due to the bonds in metal oxides. The varying widths of the bands demonstrate the presence of bands in both the SmF sample and the spinel ferrites MF. There are two frequency bands (ν1 and ν2); the frequency band (ν1) is for the stretching vibration at the tetrahedral (A) site of the spinel structure, which has a high frequency. The second frequency band (ν2) is located in the lower frequency range and corresponds to two groups: one for the octahedral (B) sites in spinel structures and the other for O-Fe-O perovskite structures. Additionally, the band at 630 cm^− 1^ was attributed to the stretching band of cadmium oxide ^27^, which agrees with the XRD results. Table 3 displays the bands for these investigation samples compared with similar samples; there are slight changes compared to the literature ^26–31^. This shows that an interaction between the MF and SmF affects the ion vibration in the crystal lattice in all of the nanocomposites that are being studied. To put it differently, the force between MF and SmF modifies the ion vibrations within the interstitial sites (A and B-sites), resulting in modifications to the lattice parameter and its characteristics. Fe–O bands typical of both perovskite and spinel phases will be visible in the SmFeO₃–MFeO₄ nanocomposites, even with shifts due to interfacial strain, small lattice distortions, and cation redistribution between tetrahedral and octahedral sites, which can all cause a modest shift in the location of the Fe–O stretching bands from both SmF and MF. Whereas broadening of the band at the nanoscale and increased surface disorder and strain broaden the Fe–O absorption peaks. This broadening is stronger at interfaces where perovskite and spinel lattices meet. Changes in peak intensity may be the result of merged peaks brought on by interfacial interactions, cation redistribution, and nanoscale effects, or they may represent changed dipole moment strengths of Fe–O vibrations at the composite interface. Furthermore, in the SmF sample, the peaks at 1384 and 1491 cm⁻¹ represent C-H stretching vibrations. Due to the mutual force between SmF and MF, these peaks shifted to the range of 1428–1565 cm⁻¹ in the composite samples.

**Table 3:** FTIR bands comparative between our work and others.

Sample	ν1	ν2	Ref.
**SmF**	411	542	**Our work**
**SmF**	416	551	**28**
**NiF/SmF**	413,424	542,600	**Our work**
**NiF**	435	601	**29**
**NiF**	399	598	**30**
**CoF/SmF**	415	533	**Our work**
**CoF**	482	605	**31**
**CoF**		547	**32**
**ZnF/SmF**	408,423	531	**Our work**
**ZnF**	-	532	**33**
**CdF/SmF**	418,436	544,631	**Our work**
**CdF**	480	545	**34**

### Optical properties

**Fig. 6:**
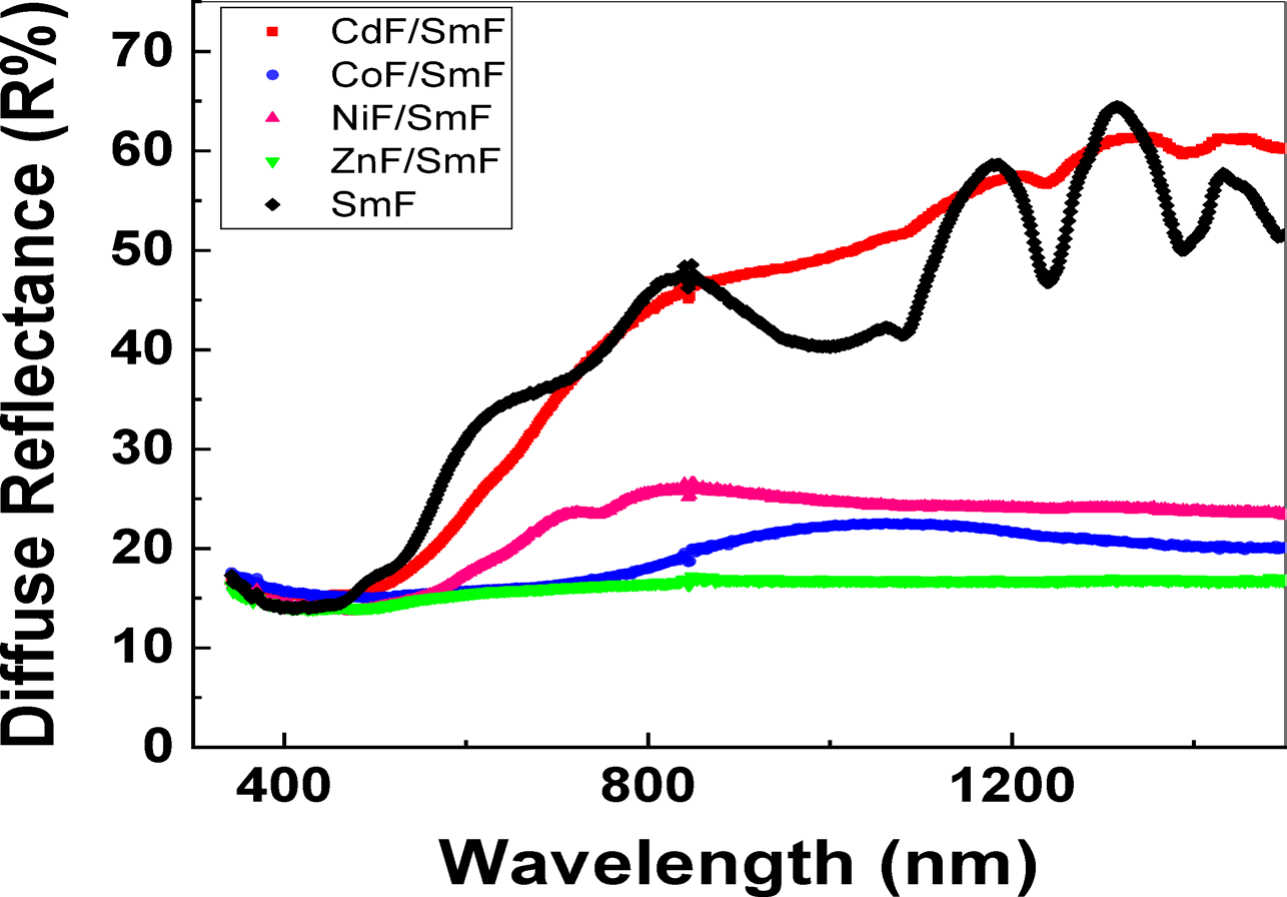
Optical reflection spectra for SmFeO_3_ and MF/SmFeO_3_ nanocomposites.

**Fig. 7:**
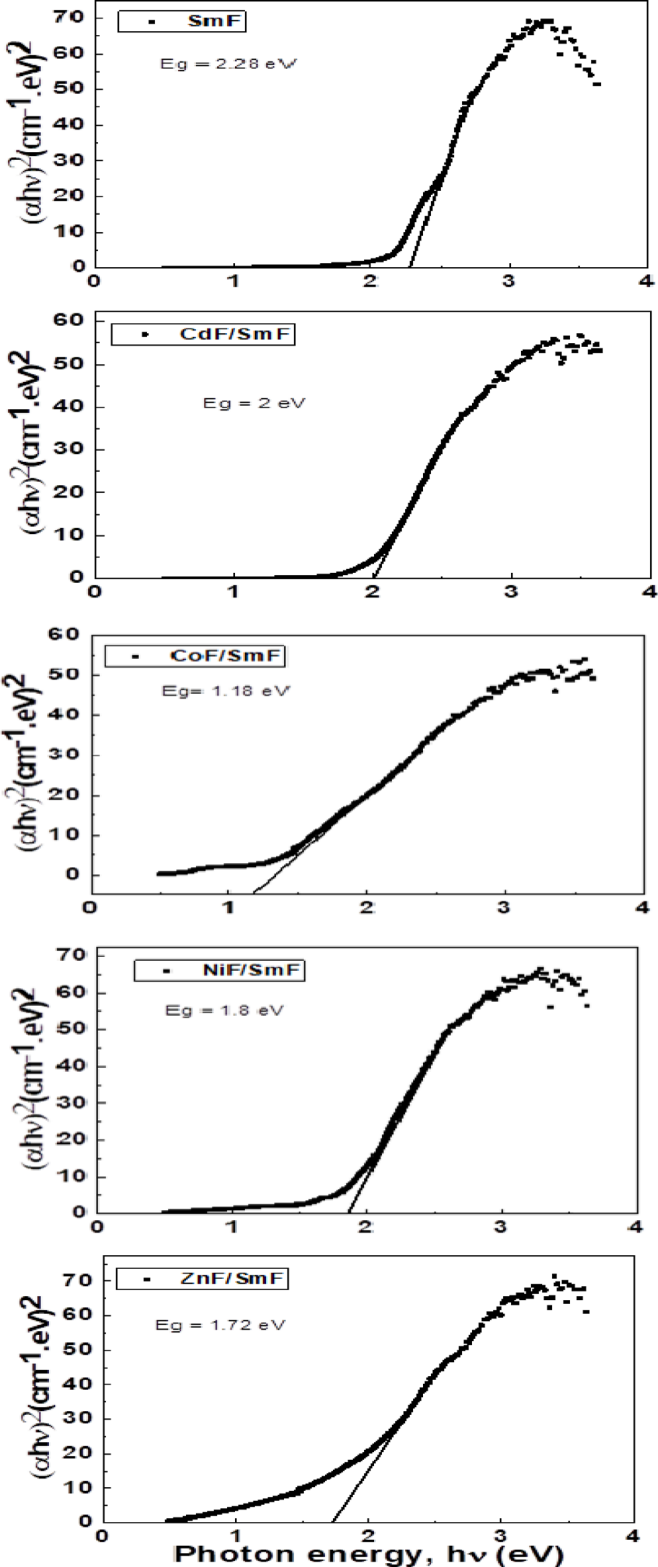
Optical band gap for SmFeO_3_ and MF/SmFeO_3_ nanocomposites.

The optical behavior and energy band gap of MF/SmF nanocomposites were examined using UV-visible absorption spectroscopy at room temperature, in the wavelength range of 300–1500 nm, as shown in Fig. 6. The energy-dependent absorption coefficient α can be expressed by the following Eq. (1):

1 $$(\alpha\:h\:\nu)^{1/\gamma}= B (h\nu-Eg)$$

where h is the Planck constant, ν is the photon’s frequency, Eg is the band gap energy, and B is a constant. The γ factor depends on the nature of the electron transition and is equal to 1/2 or 2 for the direct and indirect transition band gaps, respectively ^35^. The band gap energy is usually determined from diffuse reflectance spectra. The Tauc plot of (hν)^2^ versus hν is used to generate a direct energy bandgap of MF/SmF nanocomposites in the linear region of the Tauc plot, as illustrated in Fig. 6. According to Fig. 7, the band-gap energy of the SmF sample is 2.28 eV. The CoF/SmF composite’s calculated E_g_ value is 1.18 eV, and the NiF/SmF composite has an energy band gap of 1.8 eV. Additionally, because of the ZnF/SmF composite’s 1.72 eV band gap, we are motivated to look into its possible use in heterogeneous photocatalysis when exposed to visible light ^33–35^. This implies that by merely altering the spinel ferrites, the band gap can be readily controlled across a broad range of energies. The CdF/SmF composite’s energy band gap shrank to 2 eV. This band gap decreases due to the broadening of the energy band (the difference in energy between the valence band and the conduction band) caused by the larger particles. The decline may be due to new energy levels near the conduction band (CB). Particularly, anions significantly affect the band-gap of perovskites based on rare earth elements.

Table 4 shows that the energy band gap values of MF/SmF nanocomposites differ from recent literature ^36–41^. The quantum confinement effect may have changed the band gap of the nanocomposite sample in comparison to existing literature ^33^. Therefore, the variations in shape, particle size, and the type of added oxide all influence the energy gap variation.

**Table 4:** Band gap comparative between our work and others.

Sample	Eg	Ref.
**SmF**	2.28	**Our work**
**SmF**	2.01	**36** **36**
**CuO(10 wt%)/SmF**	1.81	
**NiF/SmF**	1.8	**Our work**
**NiF**	1.51	**39** **39**
**SnS**_**2**_ **/NiF**	1.74	
**NiF**	1.59	**40**
**NiF/[(0.1)MnO-0.9CoO]**	1.13	
**CoF/SmF**	1.18	**Our work**
**CoF**	1.7	**41** **41**
**CoF/ZnO**	3.3	
**CoF**	1.76	**42**
**ZnF/SmF**	1.72	**Our work**
**ZnF**	2.11	**43** **43**
**ZnF-TiO**_**2**_	2.3	
**CdF/SmF**	2	**Our work**
**CdF**	3.27	**44** **44**
**NiO/CdFe**_**2**_ **O**_**4**_	2.98	

### Magnetic hysteresis loops (VSM)

**Fig. 8:**
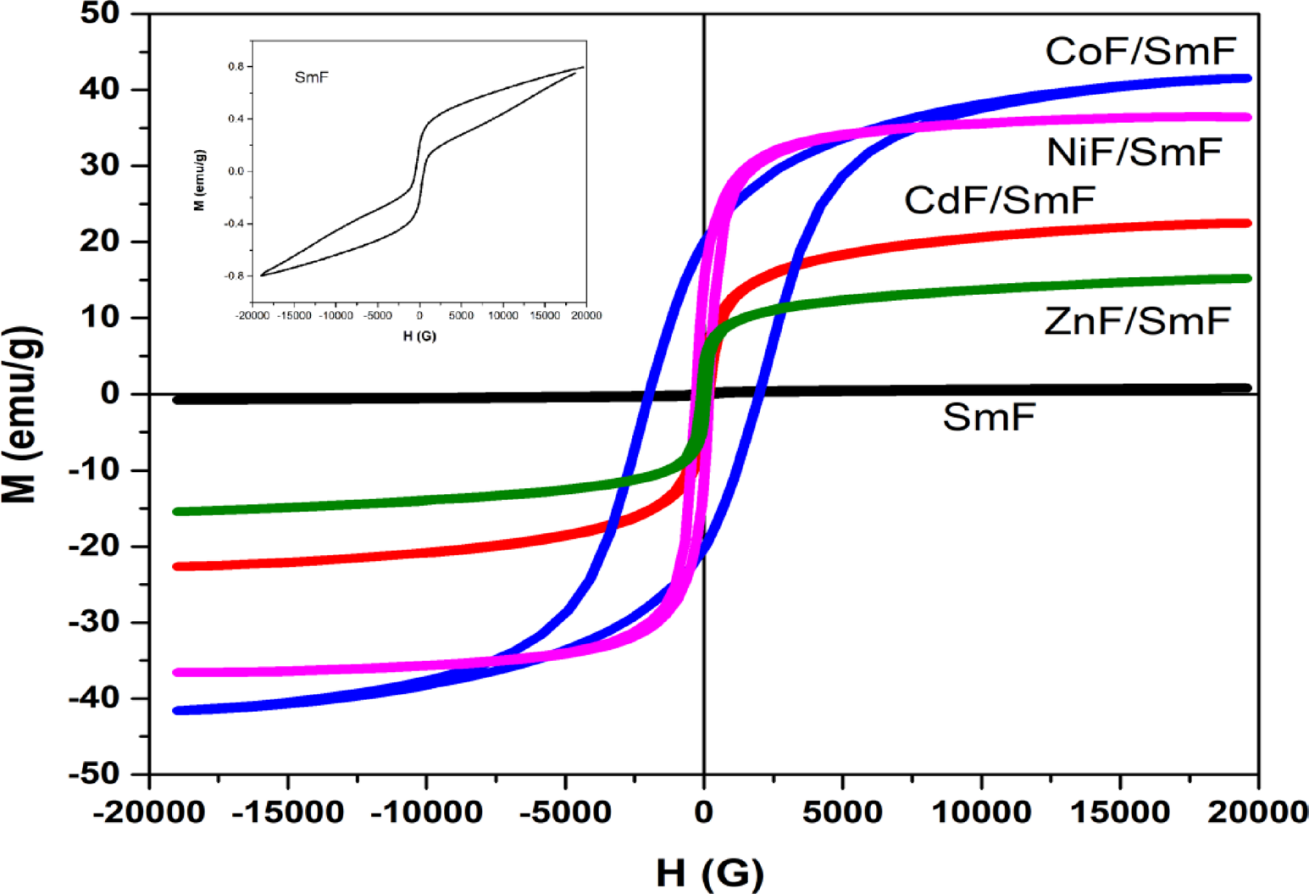
M-H curves for SmFeO_3_ and MF/SmFeO_3_ nanocomposites.

Several factors influence the magnetic properties of ferrites, such as cation distribution, crystal size, morphology, and chemical composition. At the nanoscale, the change in the cation distribution within the ferrite leads to the formation of vacancies; consequently, these variations influence the magnetic properties of the material. Figure 8 illustrates the hysteresis loops for the MF/SmF nanocomposites at room temperature. Inset Fig. 8 shows that the SmF sample exhibits a hysteresis loop of a saturation magnetic moment of 0.796 emu/g at 20 KOe. Due to its canted spin structure [23 and 45], SmF exhibits a residual magnetic moment (0.209 emu/g), which contributes to its weak ferromagnetism while maintaining an antiferromagnetic G-type magnetic structure resulting from the Fe^3+^ electron spins in the rhombohedral distorted perovskite. In other words, SmF has a coercive magnetic field (505.8 Oe) and remanent magnetization (0.209 emu/g) resulting from its weak antiferromagnetic nature and residual magnetic moment from a canted spin structure. For the SmF, six O^2−^ ions surround the Fe^3+^ ion, and two adjacent FeO_6_ octahedral shares an O^2−^ ion peak. The interaction between SmF and MF may cause the octahedral to tilt and create a canted shape. These results are very similar to those of Abeer Alshoekh ^23^.

As shown in Table 5, the squareness ratio of all samples (Mr/MS) is below 0.5, indicating the presence of multi-domain grains. The behavior of magnetic particles is influenced by their size, which allows us to classify them into two distinct categories: single-domain (SD) and multi-domain (MD). Single-domain grains exhibit magnetic hardness, characterized by elevated coercivity and remanent magnetization. In contrast, multi-domain grains, which are larger, tend to have more complex magnetic structures that can lead to lower coercivity and reduced remanent magnetization, making them less stable in their magnetic properties. Also, multi-domain particles contain domain walls, where coercivity generally decreases as particle size increases. The CoF/SmF particles, measuring 33 nm, are likely single domain, exhibiting a high coercivity of 1941 G at room temperature with a high magnetocrystalline anisotropy constant. Conversely, the other MF/SmF nanoparticles appear multi-domain (as shown in Table 5).

**Table 5:** VSM parameters.

Sample	SmF	NiF/SmF	CoF/SmF	ZnF/SmF	CdF/SmF
**Ms (emu/g)**	0.796	36.526	41.584	15.3	22.571
**Hc (G)**	505.8	172.9	1941	63.188	114.6
**Mr (emu/g)**	0.209	10.87	19.747	2.616	3.672
**S**	0.262	0.298	0.475	0.179	0.163
**K**	419.39	6578.5	84,078	1007.1	2694.4
**µ**_**B**_	0.0362	3.1953	3.6396	1.3568	2.1918

The Bohr Magneton (µ_B_), magnetocrystalline anisotropy constant (K), and initial permeability (µ_i_) are listed in Table 5 using the formulas provided in reference ^46^.

2 $${\rm \upmu\:B = [M \times Ms] / [5585 \times \uprho\:XRD]}$$

3 $${\rm K = [H_{c} \times M_{s}]/ 0.96}$$

4 $$\upmu\:i = [M\frac{2}{s}\times D] / K$$

Where the M, M_s_, H_c_, and D, are the molecular weight, magnetic saturation, coercivity, and grain size of the MF/SmF nanocomposites, respectively. Magnetocrystalline anisotropy, the Bohr magneton, magnetic domains, and superexchange interactions are all influenced by changes in saturation magnetization (Ms), remanent magnetization (Mr), and coercivity (Hc). Other studies ^47,48^ have also reported a similar dependence of coercivity on variations in the magnetic moment. Due to decreased anisotropy barriers, the coercive field typically reduces as particle size decreases ^[47-49]^.

All the MF/SmF samples exhibited saturated magnetic hysteresis loops clearly, confirming their intrinsic ferromagnetic nature. The magnetization of the samples varies based on the ferromagnetic characteristics of the MF. Consequently, the interaction between SmF and MF exhibits distinct ferromagnetic properties. The Fe at the B site of MF, as indicated in Table 2, with the heavy Sm ion in SmF, significantly enhances the magnetic interaction between MF and SmF. The FeO_6_ octahedron of the Fe–O–Fe bond angles tilts due to deformation caused by this structure ^45^ and ^50^. Consequently, the inclusion of SmF into the MF system profoundly transforms its magnetic properties, making this combination particularly impactful. To validate this, Table 6 provides a comparison of the magnetic properties of the SmF/MF with those reported in previous journals.

**Table 6:** Magnetic parameters comparative between our work and others.

Parameter	Ms (emu/g)	Hc (Oe)	Mr (emu/g)	Ref.
SmF	0.796	505.8	0.209	Our work
SmF	1.51	200	0.485	25
SmF/NiF	36.526	172.9	10.87	Our work
NiF	18.342	218.59	5.0198	29
NiF@MgF	32.5	153	8.7	20
SmF/CoF	41.584	1941	19.747	Our work
CoF	79.57	1001.12	28.96	2
BaZr_0.4_Ti_0.6_O_3_/CoFe_2_O_4_	19.35	1010	7.19	37
SmF/ZnF	15.3	63.188	2.616	Our work
ZnF	5.6	9.6	0.057	22
ZnF@MgF	17.7	75.7	2.3	20
SmF/CdF	22.571	114.6	3.672	Our work
CdF	23.34	153	-	5
0.5NiO/0.5CdFe_2_O_4_	1.28	20.17	6.07	44

### **Electron paramagnetic resonance (EPR)**

**Fig. 9:**
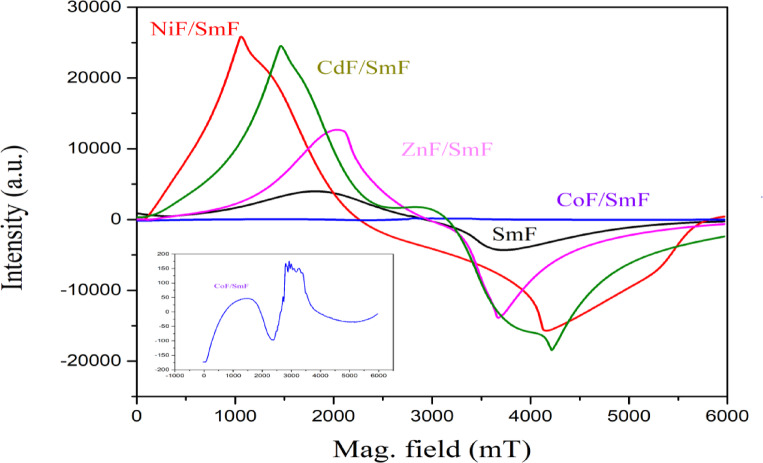
EPR spectra for SmFeO_3_ and MF/SmFeO_3_ nanocomposites.

Figure 9 displays the EPR spectra of the MF/SmF samples, which show broad features, asymmetrical signals. All samples exhibit unpaired electrons and show magnetic behavior. Each sample displays different intensity responses, with significant changes observed in the intensity peaks. Using the formula developed by Poole and Farach ^51^ g-factor of all MF/SmF can be calculated:

5 $$\:g=\frac{h\nu\:}{{\mu\:}_{B}{H}_{r}}$$

Where υ = frequency of electromagnetic radiation, h (ℏ) = Planck’s constant, µ_B_ = Bohr magneton, ***Hr*** = resonance field. Three parameters that characterize the EPR spectra: peak-to-peak line width (ΔHpp), resonant magnetic field (Hr), and g-factor shown in Table 7. It is evident from Table 7 that the values of ΔHpp and the g-factor vary with the magnetic field (MF). In ferrites, these changes in ΔHpp and the g-factor can be attributed to dipole-dipole interactions and superexchange interactions ^52^. Essentially, the characteristics of the Electron Paramagnetic Resonance (EPR) signal, including its width and the observed resonance, primarily result from the superexchange interactions between Me³⁺ and Me²⁺ in the magnetic field. The g-factor is large than the free electron (2.0023) because of the strong dipolar interactions caused by the highly agglomerated nanoparticles. Theoretically, g values indicate the super-exchange or magnetic dipolar interactions between magnetic ions. The g value will be higher if the dipolar interactions predominate. Conversely, a stronger super-exchange will result in a lower g value. The g values of 2.32 for CdF/SmF and 2.44 for ZnF/SmF are attributed to the exchange-coupled Fe³⁺ ion in distorted octahedral and tetrahedral sites, which dominated the super-exchange of Fe^3+^-O-Fe^3+ ^^53^. This observation agrees with the cation distribution from the XRD analysis and with other literature [54, and 55]. On the other hand, the NiF/SmF and CoF/SmF have high value of g-factor 3.26 and 2.78 respectively; this indicates the magnetic dipolar interactions between magnetic ions at A- and B-sites. Additionally, The EPR signal provides valuable insights into magnetic behavior and crystal defects. The g value is affected by micro magnetic structures and local symmetry variations within the crystal^56^. The deformation of the MF/SmF spectra is due to the interaction between MF and SmF, so all compounds have differing g-factors, and the intensity of the signal is different.

**Table 7:** EPR parameters.

Sample	SmF	NiF/SmF	CoF/SmF	ZnF/SmF	CdF/SmF
**Hr (mT)**	292.93	212.56	248.63	283.52	298.19
**ΔΗ(µΤ)**	186.56	307.51	67.71	159.68	275.09
**Hin (mT)**	57.07	127.44	91.37	56.48	41.81
**g (factor)**	2.45	3.26	2.78	2.44	2.32

Table 7 also shows that the resonance field (Hr) is lowest for NiF nanoparticles and increases gradually for CoF, ZnF, and SmF, reaching its highest value for CdF nanoparticles, and are different from the resonance field value (340 mT) for a free electron. In contrast to EPR spectroscopy, where ν remains constant, the resonance magnetic field should decrease as the g-factor rises (Eq. 5). The decrease in the magnetic field (Hr) is due to crystalline isotropy and increased demagnetization. When SmF ions are added to MF compounds, they enhance super-exchange interactions. This increase in interactions leads to a rise in the internal magnetic field and a decrease in the resonance magnetic field ^54^. This trend of dH, f-factor, and Hr values of CoF ZnF, and CdF was observed in other studies ^[57-59]^. Several factors can influence the magnetic field strength (Hr), including anisotropy, porosity, inhomogeneous magnetization, saturation magnetization, and the internal magnetic field (Hin) ^43^. Among these factors, the internal magnetic field plays the most critical role. Hin is due to demagnetization, dipole-dipole interactions between nanoparticles, and the internal magnetic field of agglomerates. Consequently, the effective magnetic field (Heff) is equivalent to the sum of two components ^60,61^.

6 $$\:{H}_{eff}={H}_{r}+{H}_{in}$$

It is evident from Table 7 that as the internal field rises, the resonance field falls and vice versa. Thus, the mutual force between them influences the magnetic parameters of MF and SmF. This interaction causes changes to the values of Lande’s g-factor, the peak-to-peak line width (ΔHpp), and Hr. Therefore, by using the values of Lande’s g-factor and peak-to-peak line width (ΔHpp), one can understand the magnetic behavior of MF/SmF. Consequently, the mutual force can change the magnetic behavior of two combined compounds, consistent with other studies ^[62-65]^.

## Conclusion

The nanocomposites MF/SmF were synthesized using a two-step sol-gel process. The XRD analysis was successful and revealed the presence of orthorhombic with a cubic spinel structure without any secondary phase; thus, the combination of MF and SmF demonstrated success. The effect of the mutual force between MF and SmF was evident; the unit cell volume of SmF with MF is lower than in the single phase without MF. This reduction can be attributed to the pressure from the spinel phase in the composite. The microstrain values of MF/SmF tend to increase to those of pure SmF. The cation distribution for NiF and CoF exhibits partial inverse spinel characteristics, while CdF and ZnF are classified as normal spinel. FTIR spectra have absorption of two fundamental bands (υ1 and υ2), which are typically visible in normal and inverse cubic spinel phases. Fe–O bands typical of both SmFand MF suffer from shifts due to interfacial strain, small lattice distortions, and cation redistribution between tetrahedral and octahedral sites. The findings from optical investigations on SmF indicate that MF/SmF nanocomposites have a small optical band gap of 2.28, 1.8, 1.18, 1.72, and 2 eV, which leads to different optical properties. Therefore, these materials may be the best photocatalysts in visible light.

The hysteresis loop of SmF exhibits a residual magnetic moment (0.209 emu/g), contributing to its weak ferromagnetism. The magnetic hysteresis loops reveal and confirm their intrinsic ferromagnetic nature. The magnetization of the nanocomposites varies based on the ferromagnetic characteristics of the MF. The CoF/SmF (33 nm) is typically a single domain with a high coercivity of 1941 G at room temperature, with a significant magnetocrystalline anisotropy constant (84078). In contrast, other MF/SmF nanoparticles are generally multidomain. The EPR spectra of the samples MF/SmF exhibited broad, asymmetrical signals. The g values for CdF/SmF (2.32) and for ZnF/SmF (2.44) are due to the exchange-coupled Fe³⁺ ion being observed in both distorted octahedral and tetrahedral sites. On the other hand, the NiF/SmF and CoF/SmF have high values of g-factor, 3.26 and 2.78, respectively; this indicates the magnetic dipolar interactions between magnetic ions at A- and B-sites. The resonance field (Hr) is lowest for NiF nanoparticles, and it increases gradually for CoF, ZnF, and SmF, reaching its highest value for CdF nanoparticles. By incorporating SmF ions into MF compounds, the super-exchange interactions have been enhanced, which increases the internal field and reduces the resonance magnetic field. The CoF/SmF exhibits single-domain grains with high coercivity and remanent magnetization, making it magnetically hard. The NiF/SmF possesses multidomain region domain walls and high Ms values, resulting in the highest value of Hin. The CdF/SmF and ZnF/SmF have multidomain regions, resulting in a lower value of Hin.

## Data Availability

The generated datasets and/or analyses during this study are available, and the relevant information needed for the manuscript with the corresponding author is available upon reasonable request.
